# Pharmacokinetic and pharmacodynamic analyses of nafamostat in ECMO patients: comparing central vein and ECMO machine samples

**DOI:** 10.3389/fphar.2025.1541131

**Published:** 2025-05-23

**Authors:** Dong Hwan Lee, Jae Ha Lee, Ji Hoon Jang, Yong Kyun Kim, Gaeun Kang, So Young Jung, Minyoung Her, Hang Jea Jang

**Affiliations:** ^1^ Department of Clinical Pharmacology, Hallym University Sacred Heart Hospital, Hallym University College of Medicine, Anyang, Republic of Korea; ^2^ Department of Internal Medicine, Inje University Haeundae Paik Hospital, Inje University College of Medicine, Busan, Republic of Korea; ^3^ Division of Infectious Diseases, Department of Internal Medicine, Hallym University Sacred Heart Hospital, Hallym University College of Medicine, Anyang, Republic of Korea; ^4^ Division of Clinical Pharmacology, Chonnam National University Hospital, Gwangju, Republic of Korea; ^5^ Department of Dermatology, Inje University Haeundae Paik Hospital, Inje University College of Medicine, Busan, Republic of Korea; ^6^ Division of Rheumatology, Department of Internal Medicine, Inje University Haeundae Paik Hospital, Inje University College of Medicine, Busan, Republic of Korea

**Keywords:** nafamostat mesylate, extracorporeal membrane oxygenation, pharmacokinetics, pharmacodynamics, nonlinear mixed effect model, turnover model, activated partial thromboplastin time, Monte Carlo simulation

## Abstract

**Objectives:**

To better understand nafamostat mesylate (NM) dose requirements during extracorporeal membrane oxygenation (ECMO), this study investigated its pharmacokinetic/pharmacodynamic (PK/PD) properties by comparing samples from the systemic circulation of patients and from the ECMO circuit. It specifically examined the relationship between NM concentration and activated partial thromboplastin time (aPTT) changes, aiming to provide a foundation for future dosing optimization.

**Methods:**

In this prospective study, 24 ECMO patients received a continuous infusion of NM through a dedicated stopcock located before the ECMO pump. This placement targets the anticoagulant effects of NM specifically to the ECMO circuit without substantially affecting the patient’s overall coagulation status. The starting dose was 15 mg/h, adjusted to keep the aPTT within a target range of 40–80 s. Blood samples were collected from both the patient’s central venous catheter and the ECMO circuit for PK/PD analysis using a nonlinear mixed effects model.

**Results:**

The PK profiles of NM, derived from samples taken from both the patient’s catheter and the ECMO circuit, were best described by a two-compartment model. In the PK/PD models, the effect of NM on prolonging aPTT was described using a turnover model. NM was shown to inhibit the decrease in aPTT in the turnover model. In the patient model, the maximum inhibitory effect (Imax) of NM on the reduction of aPTT was 35.5%, and the concentration of NM required to achieve half of this maximum effect (IC50) was 350 μg/L. On the other hand, in the ECMO model, the Imax for aPTT reduction was 43.6%, with an IC50 of 581 μg/L.

**Conclusion:**

The PK/PD models developed from samples collected from both the patient and the ECMO circuit indicate significant differences in PD. Given the observed variability and the high risk of bleeding in ECMO patients, a predictive model incorporating these differences and patient-specific variables could significantly improve anticoagulation management.

## 1 Introduction

Extracorporeal membrane oxygenation (ECMO) is a life-support technique used in critical care medicine to provide temporary support to the lungs and heart of patients with severe respiratory or cardiac failure (Murphy et al., 2015). Veno-venous (VV) ECMO provides both oxygenation and carbon dioxide removal by draining deoxygenated blood from a vein, oxygenating it externally, and returning it to the patient’s circulation through another vein. Veno-arterial (VA) ECMO, on the other hand, supports both oxygenation and cardiac output by draining deoxygenated blood from a vein and returning oxygenated blood to the patient’s circulation through an artery (Abrams et al., 2014; Gajkowski et al., 2022). The use of ECMO has increased rapidly in recent years due to advances in machinery and the increasing demand. The Extracorporeal Life Support Organization (ELSO) is a non-profit organization founded in 1989 and has been registering patient data on the use and outcomes of ECMO ever since. By 1 July 2016, a total of 78,387 patients had been registered, increasing to 196,108 by 2022 (Thiagarajan et al., 2017; Extracorporeal Life Support Organization, 2023). In 2021, during the height of the coronavirus disease 2019 (COVID-19) pandemic, a total of 21,895 patients were enrolled across 591 centers.

Anticoagulation therapy is a crucial requirement for patients undergoing ECMO due to their heightened risk of thrombotic complications from coagulation pathway activation and blood exposure to foreign surfaces during treatment (Murphy et al., 2015). Anticoagulants, including unfractionated heparin, argatroban, and bivalirudin, are utilized for antithrombotic therapy during ECMO. Therapeutic monitoring of these anticoagulants involves various parameters such as activated clotting time, activated partial thromboplastin time (aPTT), anti-factor Xa level, antithrombin level, and viscoelastic hemostatic assays (Levy et al., 2022; McMichael et al., 2022). Ensuring effective antithrombotic therapy to prevent thrombotic events while minimizing the risk of bleeding poses a significant clinical challenge (Cavayas et al., 2018; Nguyen et al., 2022). ELSO registry data from 2010 to 2017 were analyzed in a study, revealing that 3,044 (40.2%) of 7,570 adult patients undergoing VV-ECMO experienced bleeding or thrombus-related complications. Among these cases, 37% (1,127) had only bleeding events, 41.7% (1,270) had only thrombotic events, and 21.2% (647) experienced both (Nunez et al., 2022). Another analysis of the same registry showed that among 11,984 adult patients on VA-ECMO, 8,457 adverse events related to hemocompatibility were observed. Of these events, 62.1% (5,252) were classified as bleeding events, while 37.9% (3,205) were categorized as thrombotic events (Chung et al., 2020).

Unfractionated heparin (UFH) is widely used as the primary anticoagulant during ECMO or continuous renal replacement therapy (CRRT), given its reliable anticoagulation efficacy and extensive clinical experience (Zarbock et al., 2020; Sadeghipour et al., 2021). Nevertheless, clinical management with UFH remains challenging due to potential adverse effects such as heparin-induced thrombocytopenia, thrombotic complications, bleeding, and heparin resistance, each of which complicates patient management and necessitates careful monitoring and timely intervention. Consequently, clinicians have sought alternative anticoagulants, especially for patients prone to bleeding or those unable to tolerate UFH.

Nafamostat mesylate (NM) is a serine protease inhibitor used as an anticoagulant for ECMO or CRRT in the Republic of Korea, Japan, and China (Han et al., 2011; Baek et al., 2012; Choi et al., 2015; Kamijo et al., 2020; Lee et al., 2022). NM possesses a markedly short systemic half-life of approximately 8 min (Morikawa et al., 1983) and primarily exerts its anticoagulant activity within the ECMO circuit rather than in the systemic circulation. This pharmacokinetic (PK) profile allows targeted anticoagulation within the extracorporeal system, potentially reducing systemic bleeding risks compared to UFH, which has a significantly longer half-life of about 60 min and produces systemic anticoagulation effects. Previous clinical observations indicated that NM might lower the requirements for transfusions and reduce bleeding-related complications while maintaining similar anticoagulant efficacy as UFH within the ECMO circuit (Kotani et al., 2002; Han et al., 2011; Han et al., 2018). Furthermore, NM’s unique profile permits clinicians to selectively minimize systemic anticoagulation, and some studies have suggested that combination therapy using NM alongside low-dose UFH could prevent ECMO circuit thrombosis effectively (Yamagishi et al., 2004; Doi et al., 2020). These promising aspects of NM spurred broader evaluations of its impact on clinical outcomes like bleeding, thrombosis, and filter lifespan. A systematic review of 11 retrospective studies on patients receiving NM during ECMO revealed contrasting outcomes regarding bleeding and thrombotic events (Sanfilippo et al., 2022). Similarly, a retrospective study involving 243 patients on CRRT demonstrated that NM infusion at a rate of 10 mg/h effectively prolonged filter lifespan in high-risk bleeding patients without increasing the need for RBC transfusions or causing significant bleeding events (Baek et al., 2012). Furthermore, a prospective study involving 55 patients receiving CRRT in a high-risk bleeding state found that, among them, 31 patients received NM (NM group), while 24 did not receive anticoagulant therapy (NA group) (Choi et al., 2015). The NM group showed a significantly longer filter lifespan of 31.7 ± 24.1 days compared to the NA group, which had a filter lifespan of 19.5 ± 14.9 days (p = 0.035), while there were no differences observed between the two groups in terms of transfusion frequency and occurrence of bleeding events.

While NM is used for regional anticoagulation during ECMO and CRRT, a notable research gap exists regarding its detailed PK and pharmacodynamics (PD) in this setting. This gap hinders the development of evidence-based dosing strategies informed by PK/PD principles. The aim of this study is to develop PK/PD models using samples from both ECMO circuits and central venous catheters in ECMO patients treated with NM. This involves investigating the relationship between NM concentrations and changes in aPTT. Monitoring aPTT in patients is crucial for minimizing risks of bleeding and preventing thrombosis, while in the ECMO circuit, it specifically aids in preventing thrombus formation. This dual monitoring approach underscores the importance of precise aPTT management in optimizing therapeutic outcomes for ECMO patients.

## 2 Materials and methods

### 2.1 Patients

This prospective clinical study was conducted at Haeundae Paik Hospital, Busan, Republic of Korea, from July 2021 to October 2022. Patients older than 18 years, admitted to the intensive care unit, and receiving NM while on VV- or VA-ECMO for respiratory and/or cardiac dysfunction were eligible to participate. The ECMO system was the Permanent Life Support System (MAQUET, Rastatt, Germany). It consisted of a PLS-i oxygenator with a Bioline coating and a ROTAFLOW centrifugal pump (RF-32). The circuit was primed with 1 L of normal saline or plasma solution. The total volume of the circuit was between 500 mL and 600 mL.

### 2.2 Nafamostat dosing and sampling

NM (SK Chemicals Life Science, Seongnam, Korea; licensed by Toril Pharma, Tokyo, Japan) was continuously infused through a dedicated stopcock installed in the drainage pathway upstream of the ECMO pump. NM was started at 15 mg/h without bolus injection. The maintenance dose of NM was adjusted to achieve an aPTT range of 40–80 s. To measure the concentration of the drug, blood samples were obtained from the patient and from the ECMO circuit. Patient samples were collected from the patient’s central venous catheter, while ECMO samples were collected from the route through which oxygenated blood was infused back into the patient’s bloodstream from the ECMO oxygenator. Planned sampling times for PK model development were just before drug administration and at 3, 6, 30, 120, 300, and 480 min after the start of continuous infusion. Sampling time points were selected based on the two-compartment kinetics of NM, aiming to capture both the distribution and elimination phases. Due to clinical constraints, sampling began at the start of infusion, and time points were optimized using parameter sensitivity analysis. The total sampling duration of 6 h was intended to adequately capture the elimination phase, corresponding to approximately 3–6 times the elimination half-life (t_1/2β_), which has been reported to range from 23.1 to 120 min. Reported distribution half-lives (t_1/2α_) range from 1 to 4 min (Abe et al., 1984; Cao et al., 2008). For PD modelling, aPTT values measured immediately before dosing and at 240 and 480 min were used.

### 2.3 Nafamostat assay

NM plasma concentrations were analyzed using a liquid chromatography (LC)-tandem mass spectrometry (MS/MS) assay. The HPLC system consisted of an LC-20A system (Shimadzu, Kyoto, Japan), Kinetex XB-C18 (2.6 μm, 100 × 3.0 mm) analytical column, and Gemini C18 (4.0 × 2.0 mm) guard cartridge (Phenomenex, Torrance, CA, United States). The mobile phase consisted of A (0.1% formic acid in water) and B (0.1% formic acid in acetonitrile). The gradient run was used at a flow rate of 0.3 mL/min with an initial 10% B, which increased to 40% B until 0.1 min and held constant until 1.1 min. B was then decreased back to 10% until 1.2 min. The run time was 5 min. SCIEX Analyst software (version 1.6.3) was used for data integration. MS detection was performed using a quadrupole mass spectrometer (API4000 QTRAP system; SCIEX, Framingham, MA, United States). The analytes were detected in positive ion mode in electrospray ionization (ESI) and by Multiple Reaction Monitoring (MRM) scan mode. The MRM was carried out at m/z 174.7/166.3 for NM and 172.2/137.2 for gabapentin (IS). NM and gabapentin were purchased from Sigma-Aldrich (St. Louis, MO, United States). The standard solution of NM (1,000 mg/L) was prepared by dissolving NM in deionized water and gabapentin (1,000 mg/L) was prepared by dissolving it in methanol. Calibration standards (0.5–500 μg/L) were prepared by mixing 90 μL of blank human plasma with 10 μL of working solution (ten-fold target concentration in 50% methanol). To prepare all samples, including calibration standards, 100 μL of each plasma sample was mixed with 10 μL of internal standard solution (gabapentin at 100 μg/L in 50% methanol). Subsequently, 400 μL of methanol was added to precipitate proteins. The mixture was then vortexed for 1 min. After centrifugation at 13,400 rcf at 4°C for 2 min, the supernatant was transferred to the vial of an autoinjector and diluted 2 twofold with 20 mM ammonium acetate. Then, 5 μL of the diluted supernatant was injected into the LC-MS/MS system. From the obtained chromatogram, the ratio of the peak area of NM to that of the internal standard was calculated, and the concentration of NM in plasma was calculated.

### 2.4 Modeling and simulation

Nonlinear mixed effects modelling software (NONMEM^®^, version 7.5, ICON Clinical Research LLC, North Wales, PA, United States) was used for population PK/PD analysis. First-order conditional estimation with interaction (FOCEI) was used to estimate the fixed and random effect parameters. FOCEI allows interaction between the inter-individual variability (IIV) of the PK/PD parameters and the residual unexplained variability (RUV) of the measured observations. RUV can be by measurement error, model misspecification, or physiological variability.

For PK modelling, ADVAN1 TRANS2 and ADVAN3 TRANS4 from the NONMEM library were used to develop one- and two-compartment models, respectively. To describe the exposure-response relationship of NM over time, two kinds of models were tested: one in which NM has a direct effect on aPTT and the other in which NM affects the turnover process of aPTT. To develop the PK/PD models, the individual PK parameters were estimated using maximum *a posteriori* Bayesian estimation using the final PK model and were then added to the dataset. This ensured that the PK parameters were fixed and only the PD parameters were estimated during the development of the PK/PD model. Among the NONMEM libraries, ADVAN1 TRANS2 or ADVAN3 TRANS4 were used for PK modelling, and ADVAN6 TRANS1 was used for turnover process modelling. The PK/PD parameter was defined as θ_i_ = θ × exp (η_i_), where θ is the typical value of the PK or PD parameter, θ_i_ the individual parameter, and η_i_ the random variable associated with IIV, which was assumed to have a normal distribution with a mean of 0 and a variance of ω^2^. For the RUV, three types of error models were evaluated to best fit the data: an additive error model, a proportional error model, and a combined additive and proportional error model. Each model assumes that the residuals have a normal distribution with a mean of 0 and a variance of σ^2^. The evaluation and selection of the models were based on NONMEM objective function values (OFVs), precision of parameter estimates (relative standard errors), and diagnostic goodness-of-fit plots. In a log-likelihood ratio test, a reduction in OFV (ΔOFV) greater than 3.84 between two nested models with one degree of freedom, or greater than 5.99 with two degrees of freedom, was considered a significant model improvement. Diagnostic plots used for evaluation included conditional weighted residuals (CWRES) versus time, CWRES versus population predictions (PRED), measured concentrations versus PRED, and measured concentrations versus individual predictions (IPRED). The Perl-speaks-NONMEM software (version 5.3.1, available at https://uupharmacometrics.github.io/PsN/) was used to search for significant covariates and to evaluate the final model using a prediction-corrected visual predictive check (pcVPC) and nonparametric bootstrap method. Stepwise forward selection and backward elimination were used to identify significant covariates for PK/PD parameters, with statistical significance set at p < 0.01 (ΔOFV < −6.63 with 1 degree of freedom) for selection and p < 0.001 (ΔOFV <10.8 with 1 degree of freedom) for elimination. A covariate was considered significant if it met both the clinical relevance and the statistical significance criteria. Demographic, pathophysiological, and clinical characteristics of the patients as well as ECMO device characteristics were used in the covariate analysis. The tested demographic factors comprised gender, age, weight, and height. The pathophysiological factors included in the analysis were serum albumin level, serum protein level, serum total bilirubin level, serum creatinine level, serum c-reactive protein level, and blood urea nitrogen level. The clinical factors analyzed included primary diagnosis, presence of shock, presence of hypertension, presence of diabetes, length of stay in the intensive care unit, and duration of mechanical ventilation. The analyzed features of ECMO consisted of the duration of application, ECMO type, gas flow rate, pump speed, blood flow rate, and fraction of inspired oxygen. To evaluate the predictive performance of the model, pcVPC were conducted by comparing the 10th, 50th, and 90th percentiles of 1,000 virtual datasets generated from the final PK/PD model with the observed concentrations. The median and 95% confidence intervals for the PK/PD parameter estimates from bootstrap samples (n = 2,000) were generated to assess the stability and reliability of the model parameter estimates.

The exposure-response relationship of NM infusion rate was investigated through Monte Carlo simulations using the final patient and ECMO models. NONMEM was utilized for simulations, employing the final PK/PD parameter estimates, which included typical values, IIV, and RUV. These simulations generated NM concentrations and corresponding aPTT levels at 1-min intervals for a virtual cohort of 2,000 patients. The simulations encompassed infusion rates ranging from 10 mg/h to 50 mg/h, with 10 mg/h increments, and the infusions lasted for a duration of 6 h.

## 3 Results

### 3.1 Patients

**FIGURE 1:**
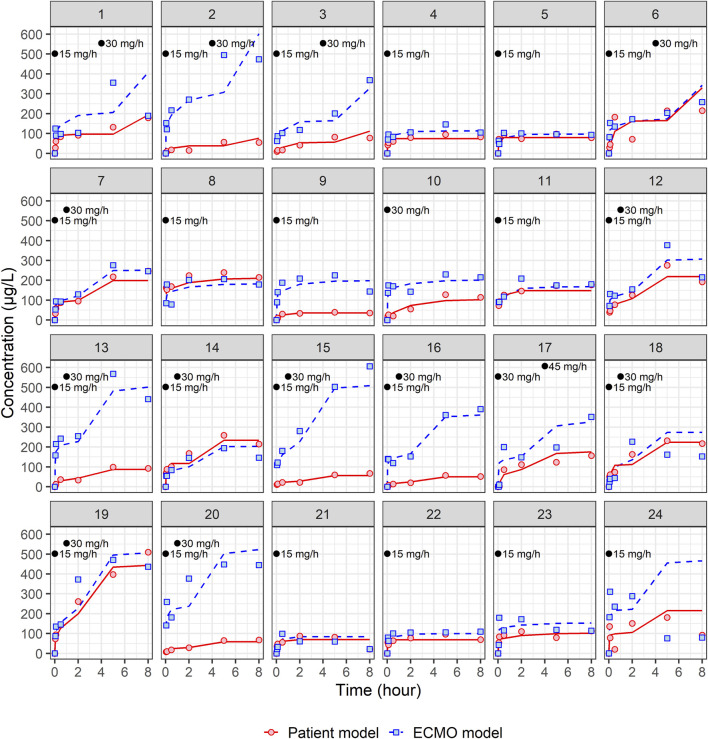
Individual fit plots for nafamostat pharmacokinetic models: comparison of observed (dot) and individual predicted (line) concentrations in patient and ECMO samples. Black dots represent the points at which nafamostat was administered along with the corresponding infusion rates.

**FIGURE 2:**
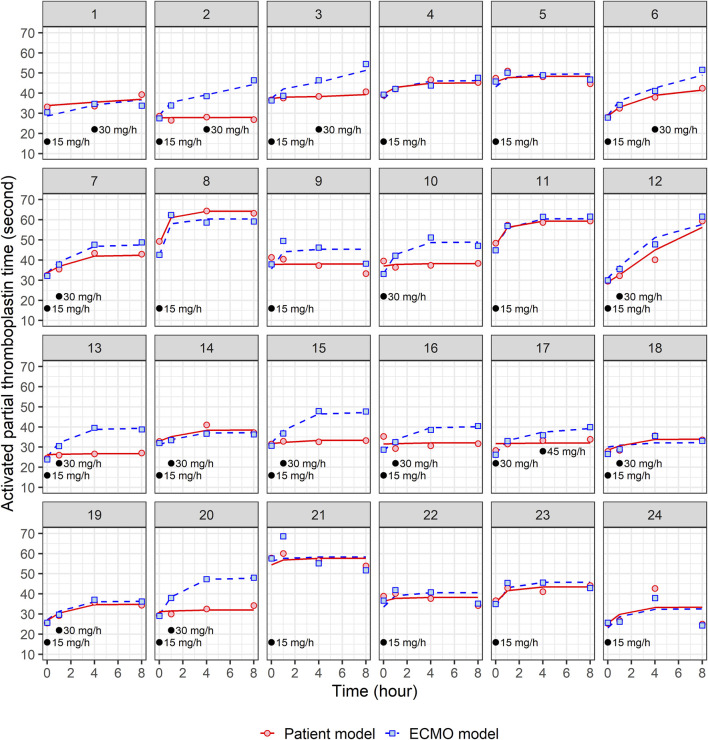
Individual fit plots for nafamostat pharmacodynamic models: comparison of observed (dot) and individual predicted (line) activated partial thromboplastin time in patient and ECMO samples. Black dots represent the points at which nafamostat was administered along with the corresponding infusion rates.

A total of 24 patients were prospectively enrolled in this study (Table 1). The primary diseases were pneumonia (n = 10), cardiogenic shock and ventricular fibrillation (n = 4), interstitial lung disease (n = 1), pulmonary thromboembolism (n = 1), aortic dissection (n = 1), gastro-intestinal infection (n = 3), and trauma (n = 4). Regarding types of ECMO employed, VA-ECMO was used in 54% of patients (n = 13) and VV-ECMO was used in 46% of patients (n = 11). Figures 1, 2 present the individual concentration–time and aPTT–time profiles, respectively, for each patient included in the study. Two patients (patients 1 and 14, 8.3%) experienced hemoptysis before the initiation of ECMO. After ECMO initiation, bleeding events were observed in six patients (25%): patient 5 (ECMO cannulation site), patient 7 (cannulation site and gastrointestinal bleeding), patient 8 (cannulation site and hemoptysis), patient 16 (cannulation site), and patients 18 and 20 (both hemoptysis). All bleeding events were classified as mild to moderate in severity. No patient required blood transfusion or developed bleeding-related shock. In Figure 1, which displays NM concentration profiles, the systemic and ECMO circuit concentrations in these patients did not deviate markedly from the rest of the cohort. For example, patients 7 and 8, despite experiencing two-site bleeding, had ECMO concentrations peaking around 150–250 μg/L and patient plasma concentrations around 200 μg/L. Patient 16, who had only mild cannulation site bleeding, maintained systemic concentrations below 100 μg/L throughout. In Figure 2, which shows aPTT profiles, systemic aPTT values in bleeding patients mostly ranged from 40 to 60 s, similar to patients without bleeding. No bleeding patient exhibited excessive systemic aPTT prolongation.

**TABLE 1:** Patient characteristics.

Characteristic	Mean (SD) or No.	Median (IQR)
Demographic characteristics		
Sex, no.	Male 17/Female 7	
Age, yr	60.3 (12.1)	61 (57.8–67.5)
Height, cm	167 (8.77)	169 (160–174)
Weight, kg	70.3 (15.7)	68.2 (62.9–76.8)
Clinical characteristics		
ICU duration, day	44.5 (62.2)	22 (12–40)
MV duration, day	40.8 (56.1)	17 (11–39.5)
Shock	Yes 13/No 11	
Hypertension	Yes 10/No 14	
Diabetes	Yes 11/No 13	
CRRT	Yes 7/No 17	
Survived	Yes 11/No 13	
ECMO characteristics		
Type	VA 13/VV 11	
Duration, day	552 (848)	186 (105–630)
FiO_2_, mmHg	0.683 (0.175)	0.7 (0.575–0.8)
Gas flow rate, L/min	4.31 (2.39)	3.75 (2.5–5.5)
Pump speed, rotation/min	2,608 (695)	2,708 (1,975–3,025)
Fluid flow rate, L/min	3.36 (1.35)	3.37 (2.49–4.37)
Laboratory characteristics		
C-reactive protein, mg/dL	12.2 (9.70)	10.4 (5.1–16)
Creatinine clearance, mg/dL	1.17 (0.87)	0.95 (0.65–1.29)
Blood urea nitrogen, mg/dL	30.2 (16.4)	26.2 (20.9–39.6)
Serum albumin, mg/dL	2.68 (0.410)	2.6 (2.4–3.03)
Total bilirubin, mg/dL	3.12 (5.62)	1.25 (0.65–3.35)
Protein, g/dL	5.10 (0.500)	5.15 (4.85–5.5)
PT, s	16.3 (3.00)	16.2 (13.7–17.7)
INR	1.45 (0.270)	1.43 (1.22–1.58)
APTT, s	50.7 (13.4)	47.2 (42.8–54.5)
Platelet count (x10^3^/μL), no.	67.3 (30.9)	64.5 (40.3–87.3)
ABGA, mmol/L	1.90 (0.930)	1.7 (1.2–2.4)

ICU, intensive care unit; MV, mechanical ventilation; CRRT, continuous renal replacement therapy; ECMO, extracorporeal membrane oxygenator; FiO2, fractional inspired oxygen; PT, prothrombin time; INR, international normalized ratio; aPTT, activated partial thromboplastin time; ABGA, arterial blood gas analysis.

### 3.2 Nafamostat assay

The lower limit of quantitation was 0.5 μg/L. The precision and accuracy of the calibration standards were 1.92%–12.58% and 89.20%–105.17%, respectively, at concentrations of 0.5, 1, 2, 5, 10, 20, 50, 100, 200, and 500 μg/L. The coefficient of determination indicating the linearity of the calibration curve over a range of 0.5–500 μg/L was greater than 0.99 for all three inter-day batches. In intra-day analysis of quality control samples, the precision was 3.87% at 2 μg/L, 2.03% at 20 μg/L, and 5.79% at 100 μg/L. The accuracy was 105.17% at 2 μg/L, 99.33% at 20 μg/L, and 104.03% at 100 μg/L. In inter-day analysis, the precision was 3.04% at 2 μg/L, 3.56% at 20 μg/L, and 1.33% at 100 μg/L. The accuracy was 99.34% at 2 μg/L, 96.67% at 20 μg/L, and 101.44% at 100 μg/L.

### 3.3 Modeling and simulation

A total of 162 patients’ central venous samples and 162 ECMO circuit samples were used to develop a population PK model for NM. The time-varying concentrations of both patient and ECMO samples were best described by the two-compartment models. The structural PK parameters for the two-compartment model were total clearance (CL), volume of distribution (Vd) for the central compartment (V1), Vd for the peripheral compartment (V2), and intercompartmental CL between V1 and V2 (Q), as indicated in Table 2. All four structural PK parameters of the patient model were estimated as larger than those of the ECMO model. In the individual fit plots (Figure 1), the concentrations in the ECMO samples were mostly higher than the concentrations in the patient samples. In the patient model, the CL was 189 L/h, and the steady-state volume of distribution (V_SS_ = V1 + V2) was 62.01 L. In the ECMO model, the CL was 85.2 L/h, and the V_SS_ was 40.63 L (Table 2). In the patient model, the V2 was significantly influenced by the gas flow rate, whereas in the ECMO model, the CL was influenced by the gas flow rate. The final PK equation of V2 in patient model is described as follows:

$$V2=\theta_{V2}\times \mathrm{EXP}\;\left(\theta_{\text{Gas}\;\text{flow}\;\text{rate}\_V2}\times \left(\text{Gas flow rate }–\;3.75\right)\right)$$

**TABLE 2:** Parameter estimates and bootstrap medians (95% confidence intervals) for final pharmacokinetic models of nafamostat in patient and ECMO models.

Parameter	Patient model			ECMO model		
	Estimate	RSE (%)	Bootstrapmedian (95% CI)	Estimate	RSE (%)	Bootstrap median (95% CI)
Structural model						
θ_CL_ (L/h)	189	14.9	182 (137–242)	85.2	7.82	85.2 (73.1–98.9)
θ_Gas flow rate_CL_				0.0999	16	0.101 (0.0603–0.139)
θ_V1_ (L)	7.01	42.5	7.50 (1.53–14.1)	3.83	17.9	3.82 (2.68–5.39)
θ_Q_ (L/h)	350^a^			46.7	46.9	46.6 (19.5–109)
θ_V2_ (L)	55.0	19.2	54.0 (28.7–114.3)	36.8	36.4	35.2 (15.9–59.6)
θ_Gas flow rate_V2_	−0.852	31.1	−0.902 (−2.48–0.4)			
Interindividual variability						
ω_CL_ (%)	61.3	13.0^b^	58.2 (32.5–73.7)	29.5	20.9^b^	27.8 (15.7–40.1)
ω_V1_ (%)	69.5^a^			62.8^a^		
ω_Q_ (%)	243^a^			134^a^		
ω_V2_ (%)	49.2^a^			0.000^a^		
Residual unexplained variability						
σ_Proportional error_ (%)	28.7	13.2	28.3 (21.3–35.9)	30.9	12.8	30.6 (23.3–38.3)

RSE, relative standard error; RSE (%) = (standard error/parameter estimate) × 100; CL, total clearance; V1, central volume of distribution; V2, peripheral volume of distribution; Q, intercompartmental clearance between V1 and V2.

^a^ fixed.

^b^ RSE (%) for standard deviation = (standard error/variance estimate) × 100/2.

The final PK equation of CL in ECMO model is described as follows:

$$\text{CL}=\theta_{\text{CL}}\times \mathrm{EXP}\;\left(\theta_{\text{Gas}\;\text{flow}\;\text{rate}\_\text{CL}}\times \left(\text{Gas flow rate }–\;3.75\right)\right)$$

Supplementary Figure S1 shows the diagnostic goodness-of-fit plots for the final PK model for patient and ECMO samples of NM. The majority of CWRES and observations were evenly distributed around the x-axis or the line of identity, which indicated that the final structural models were appropriate, and there was little bias in PK parameters. Supplementary Figure S2 displays pcVPC plots for patients and ECMO PK models. The final PK models effectively explained the observed concentrations and had good predictive performance, as the observed 10th, 50th, and 90th percentiles were mostly contained within the 95% confidence intervals of their corresponding simulated percentiles. These results suggest that the final PK models are reliable in predicting the PK parameters for NM in both patient and ECMO samples.

We developed population PD models using 95 plasma samples each from central veins and ECMO circuits, facilitating a comprehensive analysis of NM’s PD. The relationship between exposure to NM and aPTT levels over time was well explained by the turnover model. The aPTT level in the absence of NM is expressed by the mechanistic turnover equation:

$$\frac{daPTT}{dt}=Kin-Kout\times aPTT$$

where Kin is a zero-order kinetic constant that describes the mechanism by which aPTT increases, and Kout is a first-order kinetic constant that describes the mechanism by which aPTT decreases. Since there is no change in aPTT in the absence of drug (i.e., daPTT/dt = 0), baseline aPTT = Kin/Kout. The drug-induced change in aPTT will return to baseline when the drug is withdrawn.

The mechanism by which the anticoagulant effect of NM increases aPTT has been well described by the following turnover model:

$$\frac{daPTT}{dt}=Kin-Kout\times \left(1-\frac{Imax\times Cp}{IC50+Cp}\right)\times aPTT$$

where Imax represents the maximum inhibitory effect of NM, IC50 is the drug concentration that produces 50% of the Imax, and Cp is the plasma concentration of NM. No significant covariates were identified to have an impact on the PD parameters. The estimated PD parameters and the individual fit plots for the final PK/PD models for patient and ECMO models are shown in Table 3 and Figure 2, respectively. In the patient model, Imax was estimated to be 0.355 with an IC50 of 350 μg/L, while that in the ECMO model was estimated to be 0.436 with an IC50 of 581 μg/L.

**TABLE 3:** Parameter estimates and bootstrap medians (95% confidence intervals) for final pharmacodynamic models of nafamostat in patient and ECMO models.

Parameter	Patient model			ECMO model		
	Estimate	RSE (%)	Bootstrap median (95% CI)	Estimate	RSE (%)	Bootstrap median (95% CI)
Structural model						
θ_Imax_	0.355	21.8	0.356 (0.233–0.608)	0.436	9.25	0.436 (0.359–0.518)
θ_IC50_ (μg/L)	350^a^			581^a^		
θ_Kin_ (s/h)	47.5	32.5	49.7 (6.13–158)	50.6	26.9	49.3 (31.6–107)
θ_Base_ (s)	34.5	4.47	34.5 (31.9–37.8)	33.0	4.43	32.9 (30.3–36.1)
Interindividual variability						
ω_Imax_ (%)	50.7^a^			27.3^a^		
ω_EC50_ (%)	70.6^a^			0.000^a^		
ω_Kin_ (%)	105	23.3^b^	88.5 (0.000–161)	75.8		
ω_Base_ (%)	20.7	13.2^b^	20.3 (13.5–24.9)	21.2	16.4^b^	20.2 (12.9–26.8)
Residual unexplained variability						
σ_Additive error_ (s)	2.83	18.6	2.76 (1.82–3.91)	3.43	16.9	3.34 (2.03–4.41)

RSE, relative standard error; RSE (%) = (standard error/parameter estimate) × 100; Imax, maximum inhibitory effect; IC50, effective concentration of drug that causes 50% Imax; Kin, turnover rate; Base, baseline aPTT, level.

^a^ fixed.

^b^ RSE (%) for standard deviation = (standard error/variance estimate) × 100/2.

Supplementary Figure S3 shows the diagnostic goodness-of-fit plots for the final PD model for patients and ECMO samples of NM. The majority of CWRES and observed concentrations were evenly distributed around the x-axis or the line of identity, indicating a good fit between the predicted and observed values. However, there is some underprediction for early samples and overprediction for late samples in the ECMO model, indicating that the model may have some limitations in accurately predicting aPTT in certain time points of some patients. Supplementary Figure S4 shows pcVPC plots for the patient and ECMO PD models. The observed 10th, 50th, and 90th percentiles were mostly within the 95% confidence intervals of the simulated 10th, 50th, and 90th percentiles, indicating that the final PD models effectively explained the observed aPTT values and had good predictive performance. These results suggest that the final PD models reliably predict PD responses in both patient and ECMO models.

**FIGURE 3:**
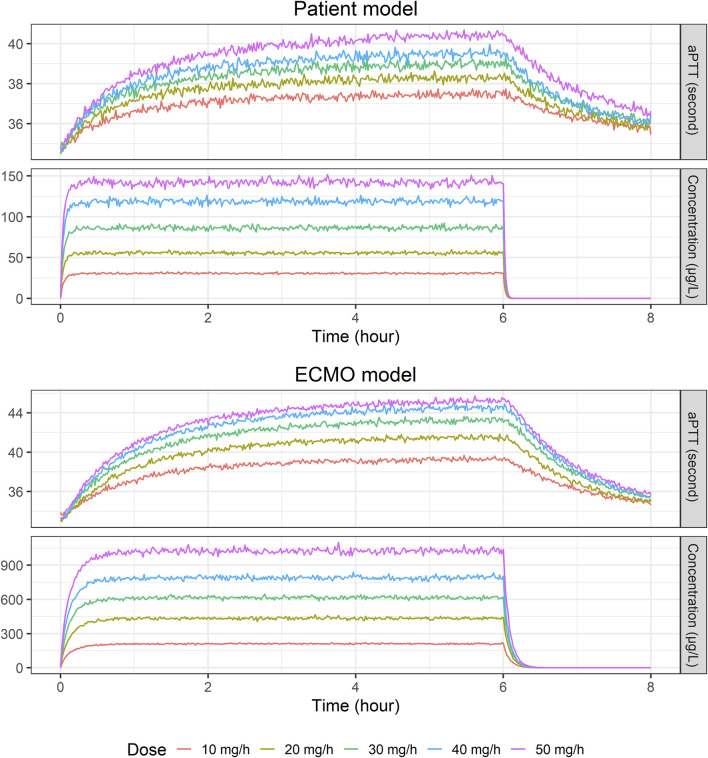
Temporal changes in median nafamostat concentration and activated partial thromboplastin time level: Insights from a simulated population of 2,000 virtual patients at various infusion rates (10, 20, 30, 40, and 50 mg/h) using final patient and ECMO models.

For the final PK/PD models, the relationship between NM exposure and aPTT level is shown in Figure 3. When NM was injected into the drainage pathway upstream of the ECMO pump at a rate of 30 mg/h, the median steady-state concentration and aPTT were approximately 88 μg/L and 39 s, respectively, in the patient model and approximately 600 μg/L and 43 s, respectively, in the ECMO model.

## 4 Discussion

Our previous study demonstrated the effectiveness and safety of NM as a regional anticoagulant in patients undergoing VA ECMO (Lee et al., 2022). We administered either NM or unfractionated heparin (UFH) and specifically compared the aPTT of blood samples obtained from the patient’s central vein with blood samples drawn from the ECMO circuit. The results revealed no statistically significant difference between the median aPTT of the patient sample (72.84 s) and the median aPTT of the ECMO sample (72.95 s) when UFH was administered. However, upon switching to NM, a significant difference was observed. The median aPTT of the ECMO sample increased to 73.13 s, while the median aPTT of the patient sample decreased to 68.42 s, with a p-value of 0.031. Moreover, when addressing bleeding adverse events, we switched from UFH to NM, resulting in significant improvement in bleeding symptoms for four patients with cannulation site bleeding, one patient with gingival bleeding, and one patient with hematochezia. Based on our previous study, we recognized the necessity for a quantitative analysis of the PK and PD of NM to enhance our understanding of its administration in ECMO patients.

To the best of our knowledge, no clinical studies have yet developed population PK/PD models for NM in patients. We developed and compared two PK/PD models, one utilizing central venous samples from patients and the other utilizing samples from the ECMO circuit. The PK profiles of NM in both sample types were well described by two-compartment models. In our study, the patient model exhibited a t_1/2α_ of 0.54 min and a t_1/2β_ of 19.7 min. On the other hand, the ECMO model demonstrated a t_1/2α_ of 1.2 min and a t_1/2β_ of 51.4 min. These results were comparable to those of other studies in Asian populations. In a Phase 1 study conducted in Japan, the observed t_1/2α_ and t_1/2β_ were 1.1 min and 23.1 min, respectively (Abe et al., 1984). A study involving healthy adults in China reported t_1/2α_ ranging from 3.65 to 3.78 min and t_1/2β_ ranging from 112.42 to 129.19 min (Cao et al., 2008). However, a study conducted with dialysis patients revealed a half-life of 8 min for NM (Morikawa et al., 1983). The advantage of remarkably short half-life of NM (approximately 8 min), especially when compared to UFH (60–90 min), argatroban (45 min), and bivalirudin (25 min), has prompted multiple studies to affirm its suitability as an anticoagulant for ECMO or CRRT patients at increased risk of bleeding (Baek et al., 2012; Lee et al., 2014; Park et al., 2015; Lang et al., 2022; Sanfilippo et al., 2022). According to our two-compartment model, following completion of dosing, the concentration declines rapidly due to the extremely short t_1/2α_. As a result, even if t_1/2β_ is prolonged, the concentration declines to very low levels during the elimination phase. However, the steady-state concentration achieved through continuous infusion may significantly differ from the steady-state concentration determined solely by a single half-life of 8 min, depending on the interplay between t_1/2α_ and t_1/2β_.

Our population PK analysis identified gas flow rate as a significant covariate influencing CL in the ECMO model and V2 in the patient model. Although a direct pharmacological interaction is unlikely, the interplay between ECMO circuit dynamics and patient hemodynamics may mediate this relationship. Gas flow rate governs CO_2_ elimination from the ECMO circuit (Schmidt et al., 2013; Strassmann et al., 2019). Enhanced CO_2_ clearance alleviates hypercapnia, which can alter hepatic perfusion, as suggested by studies showing hypercapnia affects liver blood flow (Barash et al., 2007; Cox et al., 2019). Consequently, improved hepatic blood flow could plausibly influence NM disposition, as the drug is metabolized by esterases in both the liver (carboxylesterase 2) and blood (Nakae and Tajimi, 2003). ECMO circuit dynamics also contribute significantly. In this study, NM was infused pre-pump, resulting in high initial drug concentrations within the circuit. Its moderate lipophilicity (logP ∼2) suggests potential adsorption onto circuit components, similar to other drugs like fentanyl and midazolam (Shekar et al., 2012; Gomez et al., 2022). As gas flow is often adjusted alongside ECMO blood flow in clinical practice (Strassmann et al., 2019), higher flows decrease the drug’s residence time in the circuit. This could reduce the extent of drug sequestration, impacting the observed CL in the ECMO model, a phenomenon noted with other drugs like vancomycin (Shekar et al., 2012). Systemically, the observed reduction in V2 in the patient model may reflect improved circulatory efficiency tied to enhanced gas exchange and correlated increases in blood flow, leading to less peripheral drug distribution (Ficial et al., 2021). However, the effect of gas flow on systemic CL in the patient model was not statistically significant. This is likely because patient CL is influenced by a complex array of systemic factors associated with critical illness (e.g., hepatic function, inflammation), potentially masking the isolated impact of ECMO gas flow (Tonna et al., 2021). Collectively, these plausible mechanistic links support the inclusion of gas flow rate as a relevant covariate, highlighting the intricate relationship between ECMO settings and drug disposition. Further investigation into these mechanisms is warranted. The absence of statistically significant effects on other PK parameters might be attributable to study limitations, such as sample size or confounding variables.

Among various PD models, the final turnover model provided a robust explanation for the relationship between NM concentration and the corresponding change in aPTT. This model effectively captures how NM inhibits the mechanism responsible for the decrease in aPTT. A turnover model is a mechanistic approach used to describe drug-induced indirect responses by elucidating the dynamic equilibrium between response production and response loss, providing insights into the underlying mechanisms altered by the drug (Dayneka et al., 1993; Gabrielsson et al., 2019). In our final PD model, we demonstrated the increase in aPTT by an increase in NM as a mechanism by which NM inhibits the loss of response, i.e., inhibits the decrease in aPTT. To date, there have been no studies that have modeled the relationship between NM exposure and aPTT level. However, the IC50s of our models were comparable to those of Hitomi et al. (Hitomi et al., 1985). In their study, NM, with a molecular weight of 347.37 g/mol, exhibited an IC50 value of 3.0 × 10^−9^ M (1.0421 μg/L) for plasma kallikrein inhibition and an IC50 value of 3.3 × 10^−7^ M (114.63 μg/L) for inhibiting human Hagmann factor fragment. Additionally, the concentration of NM required to double the aPTT was 5.0 × 10^−7^ M (173.69 μg/L).

When Monte Carlo simulations were performed using the final model, the PK profiles of the patient and ECMO models were significantly different, while the PD profiles were not significantly different. In a study conducted in 1972 involving adult patients not on ECMO, maintaining an aPTT within the range of 1.5–2.5 times the normal value was associated with a reduced occurrence of recurrent venous thromboembolic events (Basu et al., 1972). The current clinical recommendations suggest maintaining an aPTT level of 40–80 s during ECMO, which corresponds to 1.5 to 2.5 times the pretherapy baseline level (Sklar et al., 2016; Esper et al., 2017; Koster et al., 2019; Chlebowski et al., 2020; Sanfilippo et al., 2022). However, this recommendation has not been validated in randomized controlled trials or specifically in patients undergoing ECMO therapy (Gajkowski et al., 2022). To attain the target aPTT level, NM was administered in a dose range of 0.14–0.98 mg/kg/h (equivalent to 9.8–68.6 mg/h for a 70 kg weight) (Park et al., 2015), and in another study, at a median dose of 17.7 mg/h (range: 9.8–21.7 mg/h) (Lee et al., 2022). A systematic review of studies involving the use of NM as an anticoagulant in ECMO patients revealed that the mean dose ranged from 0.46 to 0.67 mg/kg/h (equivalent to 32.2–46.9 mg/h for a 70 kg patient) (Sanfilippo et al., 2022). Based on the findings from these studies, we conducted PK/PD simulations using 10 mg/h increments of NM infusion rates ranging from 10 mg/h to 50 mg/h. Consistent with its administration into the drainage pathway upstream of the ECMO pump, steady-state NM concentrations were markedly higher in the ECMO circuit samples compared to the patient systemic samples (Figure 3). While the observed aPTT levels might appear similar under specific conditions (Figure 3), the underlying PD models developed from patient and ECMO samples reveal important differences (Table 3). Specifically, although baseline aPTT and Kin were comparable between the two models, the IC50 was substantially higher in the ECMO model (581 μg/L) than in the patient model (350 μg/L). The Imax was also slightly higher in the ECMO model. This indicates that while the patient’s systemic circulation is inherently more sensitive to anticoagulant effect (lower IC50), it is exposed to much lower drug concentrations due to rapid metabolism and distribution. Conversely, the ECMO circuit requires higher NM concentrations to elicit a similar anticoagulant response due to its higher IC50. This interplay between higher local concentrations within the circuit and distinct local PD characteristics (higher IC50) explains how NM can achieve therapeutic anticoagulation within the ECMO apparatus while limiting excessive systemic aPTT prolongation, aligning with the goal of regional anticoagulation. In some instances, the concentrations of NM in patient and ECMO samples appear comparable, indicating that variability in carboxylesterase-mediated hydrolysis may arise from factors such as genetic variations, regulatory mechanisms at the transcriptional and posttranslational levels, and interactions with other drugs or disease states (Laizure et al., 2013; Wang et al., 2018). Traditional views hold that carboxylesterase activity is consistent among individuals; however, recent studies suggest significant variability due to genetic and environmental influences, analogous to those observed with cytochrome P450 enzymes. This emerging evidence highlights the urgent need for more comprehensive clinical studies to elucidate how carboxylesterase impacts drug metabolism and to determine its influence on the efficacy and safety of treatments (Liu et al., 2024). Similar to the differences observed between arterial and venous blood sampling in PK studies (Schaedeli et al., 2002; Fagiolino et al., 2013), the variation in NM concentrations between ECMO and patient samples can be attributed to their anatomical positions relative to the site of administration and metabolism. While ECMO samples initially reflect prehepatic exposure, the systemic circulation ensures that both compartments are subject to ongoing equilibration and hepatic clearance over time. Therefore, the observed concentration differences are consistent with physiological expectations and provide important insights into the local vs systemic distribution of NM in ECMO patients. Furthermore, additional insights into the impact of drug lipophilicity on absorption in the ECMO circuit have been provided through various studies. It was observed that lipophilic drugs exhibit significant sequestration in the ECMO circuit, with a positive correlation between lipophilicity (log P) and absorption, particularly for drugs like midazolam (0.62% recovery) and fentanyl (0.35% recovery) (Wildschut et al., 2010). Similarly, it was demonstrated that lipophilic drugs such as fentanyl and midazolam experience substantial sequestration within ECMO circuits, leading to necessitating dose adjustments during ECMO therapy (Shekar et al., 2012). With log P of NM reported to be 1.91 or 2.52 (Kim and Kim, 2022), these findings suggest that NM is likely absorbed in the ECMO circuit to a considerable extent, which would reduce the amount available to reach the patient. This may explain the observed discrepancies in NM concentrations between the ECMO circuit and patient samples. Despite these insights, the extent to which a patient’s condition influences the sequestration of NM within the ECMO circuit remains inadequately explored. This sampling site-specific analysis supports the dual clinical goals of NM therapy: preventing clot formation in the ECMO circuit while avoiding systemic over-anticoagulation and bleeding in patients. The evaluation of clinical adverse events supports the PD interpretation of NM as a regional anticoagulant. Notably, patients who experienced bleeding did not demonstrate disproportionately high NM concentrations or prolonged systemic aPTT values. These findings indicate that bleeding events were not directly attributable to NM overexposure or excessive systemic anticoagulation. Rather, they are likely to result from other clinical factors such as procedural trauma or underlying organ pathology (e.g., hemoptysis in lung disease). Furthermore, the absence of any severe bleeding cases strengthens the safety profile of NM, particularly in comparison to systemic anticoagulants like unfractionated heparin. Notably, the significant interindividual variation observed in aPTT levels between ECMO and patient samples within the same patients underscores the potential benefits of personalized treatment strategies that leverage robust models to optimally maintain aPTT levels.

ECMO has been used worldwide, and its frequency of use is increasing due to the COVID-19 pandemic. Despite technological improvement and accumulated clinical experiences, the optimal anticoagulation strategies and monitoring are not well established, and major bleeding remains both the leading cause of mortality in patients with ECMO and the Achilles heel of ECMO. In this study, we demonstrated the efficacy of NM as a regional anticoagulant comparing the PK/PD profiles of NM in both patient and ECMO samples. Additionally, the changes in aPTT level induced by NM were represented by a turnover model, where NM inhibited the decrease in aPTT. However, in real clinical practice, there is a lack of research on the actual correlation between concentration and adverse events of NM use. Considering diverse clinical situations and variables, additional research is needed to optimally adjust and titrate the dose of NM in real practice.

This study has some limitations. First, the limited number of patients hindered the identification of significant covariates, and the sampling number for each patient was insufficient for the development of a robust PD model with good predictive performance. Consequently, large between-subject variability and imprecise parameter estimates were observed. Due to these imprecise estimates, certain parameters had to be fixed to avoid further compromising the stability and reliability of the model. This highlights the need for caution when extrapolating these findings model to broader populations. Second, as shown in the individual fit plots, the model exhibited suboptimal fitting for a small subset of patients. It appeared that a few patients were documented as continuing their medication despite its discontinuation; due to the absence of any justifiable grounds for exclusion or modification of this data, they were retained for the purpose of model development. Third, we were unable to evaluate other PD markers such as activated clotting time (ACT), prothrombin time (PT), anti-factor Xa, and antithrombin activity, in addition to aPTT. Although ACT and PT data were collected, they had significant missing data and could not be used in model development. However, aPTT represents the most frequently recommended test in clinical guidelines and consensus statements. Fourth, although clinically relevant factors such as ECMO configuration (veno-venous vs veno-arterial), hemodilution, and systemic inflammation were considered during the covariate screening process, none of them demonstrated statistically significant associations with PK or PD parameters and were therefore not retained in the final model. This may be due to limited variability in these clinical characteristics or insufficient statistical power related to the sample size. Future studies with larger and more heterogeneous patient populations are needed to more definitively assess the role of these factors.

## 5 Conclusion

The PK profiles of NM in both ECMO and patient samples were well described by a two-compartment model. The changes in aPTT level induced by NM were represented by a turnover model, where NM inhibited the decrease in aPTT. Significant interindividual variability was observed in the concentration of NM and its PD effects on aPTT, underscoring the need for models that account for such variability to optimize NM dosing and achieve targeted aPTT levels. By implementing these refined PK/PD models, we might significantly reduce the risks of bleeding and thrombosis in ECMO circuits, thereby enhancing both patient safety and the overall effectiveness of ECMO therapy.

## Data Availability

The raw data supporting the conclusions of this article will be made available by the authors, without undue reservation.
