# Supplementary material for: Pharmacokinetic and pharmacodynamic analyses of nafamostat in ECMO patients: comparing central vein and ECMO machine samples
Source: Front Pharmacol. 2025 May 23;16:1541131. doi: 10.3389/fphar.2025.1541131 (PMC12141017; doi:10.3389/fphar.2025.1541131)
Supplement: Supplementary file 1 [file Supplementaryfile1.pdf]

Supplementary Material

1 Supplementary Figures

1.1 Supplementary Figures

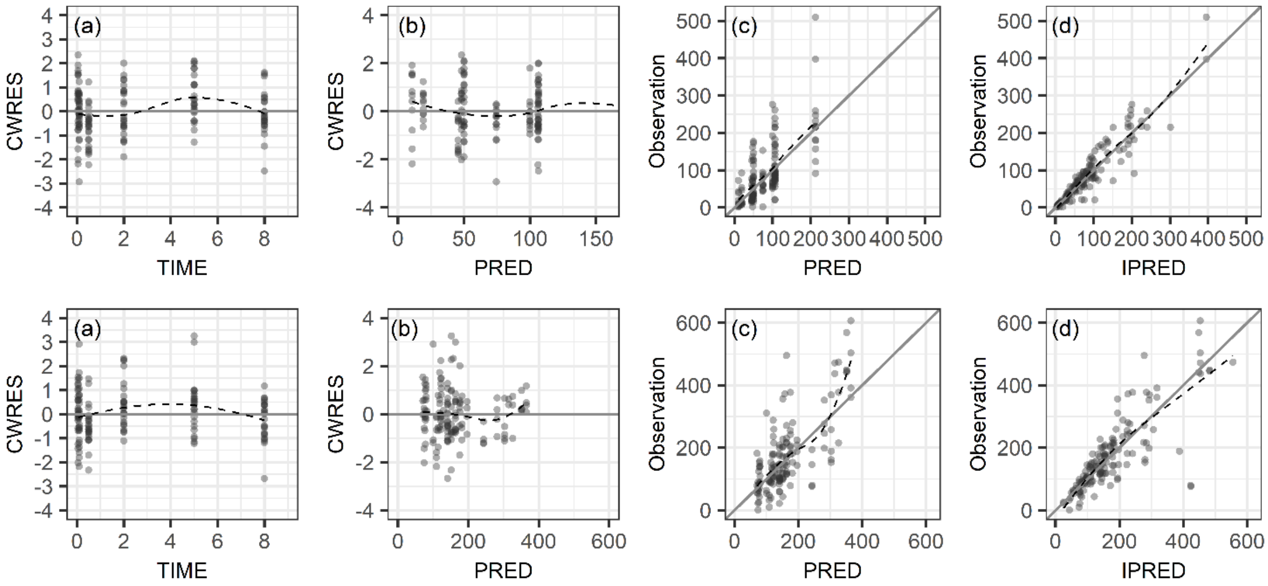

Supplementary Figure 1. Goodness-of-fit plots for final PK models for nafamostat in the patient model (top) and ECMO model (bottom): (a) and (e) conditional weighted residuals versus time, (b) and (f) conditional weighted residuals versus population predicted concentration, (c) and (g) observed concentration versus population predicted concentration, and (d) and (h) observed concentration versus individual predicted concentration. The dashed lines represent loess smoothing curves.

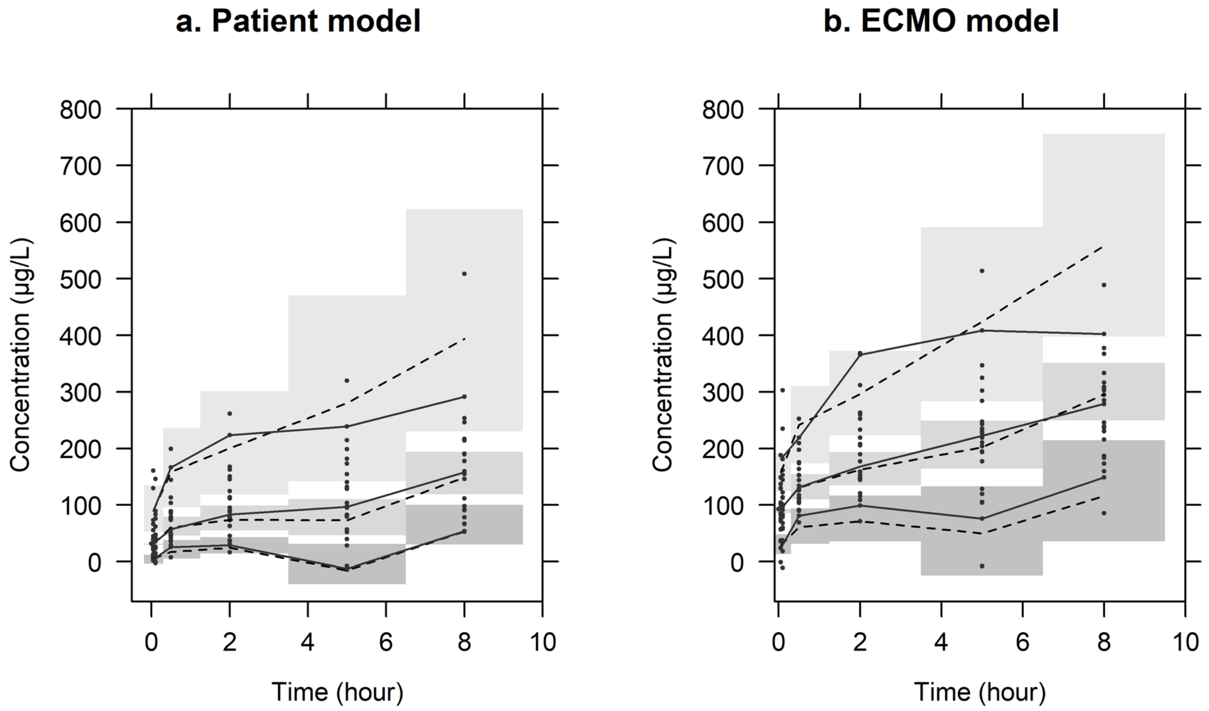

Supplementary Figure 2. Visual predictive check for final PK models for nafamostat in the patient model (a) and ECMO model (b). Closed circles, observed concentrations; solid lines, 10th, 50th, and 90th percentiles of observations; dashed lines, 10th, 50th, and 90th percentiles of simulated concentrations; shaded areas, 95% confidence intervals for the 10th (darkest gray, bottom), 50th (medium gray, middle), and 90th (lightest gray, top) percentiles of simulated concentrations.

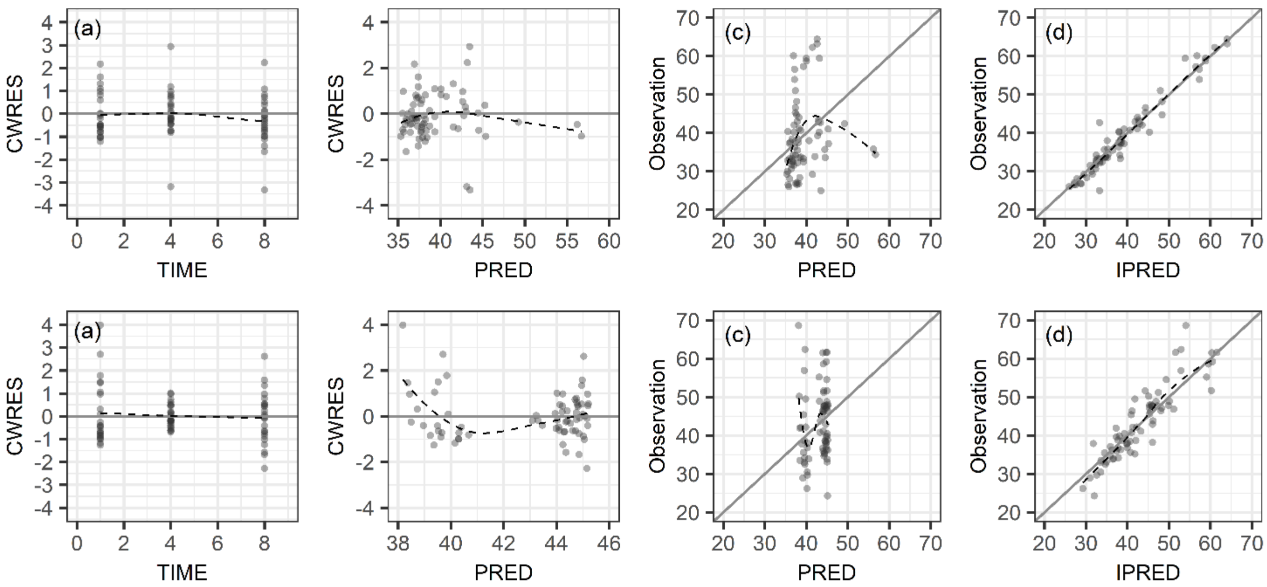

Supplementary Figure 3. Goodness-of-fit plots for final PD models for nafamostat in patient (top) and ECMO (bottom) models: (a) and (e) conditional weighted residuals versus time, (b) and (f) conditional weighted residuals versus population predicted concentration, (c) and (g) observed concentration versus population predicted concentration, and (d) and (h) observed concentration versus individual predicted concentration. The dashed lines represent loess smoothing curves.

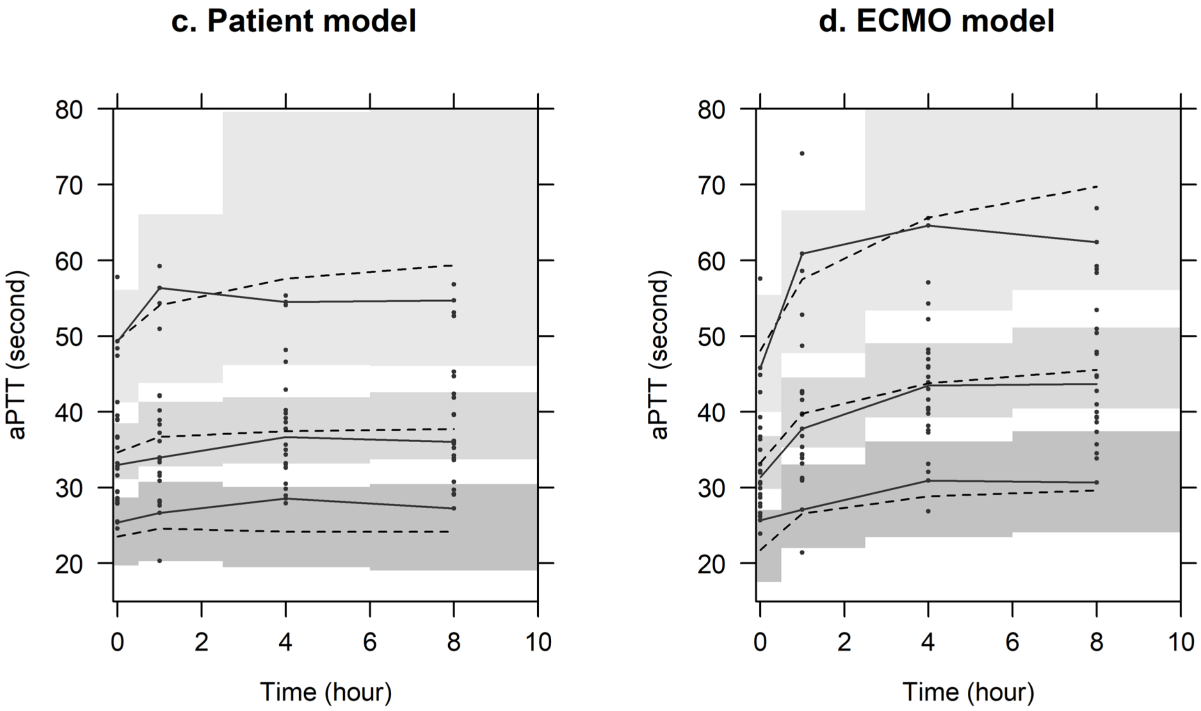

Supplementary Figure 4. Visual predictive check for final PD models for nafamostat in the patient model (a) and ECMO model (b). Closed circles, observed activated partial thromboplastin time (aPTT); solid lines, 10th, 50th, and 90th percentiles of observations; dashed lines, 10th, 50th, and 90th percentiles of simulated aPTT; shaded areas, 95% confidence intervals for the 10th (darkest gray, bottom), 50th (medium gray, middle), and 90th (lightest gray, top) percentiles of simulated aPTT.
